# Thyroxine Treatment With Softgel Capsule Formulation: Usefulness in Hypothyroid Patients Without Malabsorption

**DOI:** 10.3389/fendo.2018.00118

**Published:** 2018-03-21

**Authors:** Pierpaolo Trimboli, Camilla Virili, Marco Centanni, Luca Giovanella

**Affiliations:** ^1^Department of Nuclear Medicine and Thyroid Centre, Oncology Institute of Southern Switzerland, Bellinzona, Switzerland; ^2^Department of Medico-Surgical Sciences and Biotechnologies, Sapienza University of Rome, Latina, Italy

**Keywords:** hypothyroidism, thyroxine absorption, softgel, levothyroxine, drugs dissolution

## Abstract

**Background:**

Levothyroxine sodium (LT4) is the therapy of choice for hypothyroidism. In the last decade, new LT4 formulations, such as liquid and softgel capsules, became available. Even if some evidence has been reached in the efficacy of liquid LT4 in patients with suboptimal TSH on tablet LT4, the usefulness of softgel LT4 has been rarely studied. This study aimed at evaluating the effect of switching from tablet to softgel LT4 patients without increased need for LT4. TSH was used as proxy of LT4 bioavailability and effectiveness.

**Methods:**

During the period from April to August 2017, 19 patients on tablet LT4 treatment for hypothyroidism, mostly due to autoimmune thyroiditis, were enrolled. Subjects with causes of malabsorption or increased requirement of LT4 were previously excluded. Patients finally included were asked to switch from tablet to softgel LT4 formulation at unchanged dose and ingestion fashion (30 min before breakfast). TSH was measured with chemiluminescence immunoassays.

**Results:**

According to exclusion and inclusion criteria, 19 patients were finally selected. One of these had headache 4 days later and come back to tablet LT4, and 18 of them (16W/2M; mean age = 55 years; BMI 22.7 kg/m^2^) completed the study. They were treated with a median LT4 dose of 88 μg/day and showed a median TSH value of 3.33 mIU/L. The rate of cases with TSH ≤ 4.0 mIU/L was 61.1% (11/18 cases). When patients were re-evaluated after 3 months of softgel LT4, we observed that TSH reached levels under 4.0 mIU/L in 16/18 (88.9%) patients, TSH was lower in 11 cases, and in 6 out of 7 patients with pre-switch TSH values over the normal range. Overall, TSH values on softgel LT4 (median 1.90 mIU/L) was significantly lower from that observed during tablet LT4 (*p* = 0.0039).

**Conclusion:**

These data show that hypothyroid patients with no proven malabsorption may have an improved TSH following 3 months from the switch from tablet to softgel LT4 preparation at unchanged dose.

## Introduction

Levothyroxine sodium (LT4) is the therapy of choice for hypothyroidism (1), and the tablet LT4 is the most widely used preparation worldwide. The absorption of tablet LT4 preparation requires disintegration of the tablet and dissolution of the particles of active ingredient (2). These events are preconditions for an effective absorption of the hormone, which takes place in the small intestine (3). Its narrow therapeutic index and the deleterious effects of chronic over- and under-treatment render mandatory a fine calibration of LT4 dose (4); also, the large number of factors interfering with LT4 treatment efficacy leads, even nowadays, to the existence of a significant amount of treated patients showing TSH levels outside the therapeutic target (5). In fact, bioavailability of levothyroxine may be affected by several physiological and pathological conditions: specific habits such as dietary fiber, soy, and coffee concomitant ingestion, widespread gastrointestinal diseases such as *Helicobacter pylori*, atrophic gastritis, celiac disease, lactose intolerance, dysbiosis [see Ref. (6) for review], and widely prescribed drugs such as proton-pump inhibitors, aluminum-containing antacids, calcium carbonate, ferrous sulfate, sucralfate, raloxifene, bile acid sequestrants, and phosphate binders (7).

In the last decade, new LT4 formulations, such as liquid mono-dose ampoules and softgel capsules, became available (8). A recent meta-analysis revealed that patients with suboptimal TSH on tablet LT4 can attain a better pharmacologic homeostasis (i.e., lower serum TSH) by switching to liquid LT4 formulation at unchanged dose (9). Unlike liquid LT4, the efficacy of softgel LT4 preparation has been less investigated (10). In particular, the usefulness of softgel LT4 has been prospectively studied only in series of 8 patients with coffee-related LT4 malabsorption (11), in 31 patients with gastric diseases (12), and in 60 patients ingesting softgel capsule at breakfast (13); case reports also inquired into the efficacy of this therapeutic option in patients concomitantly treated with PPI (14), bearing gastroparesis (15) and with central hypothyroidism (16). On the contrary, there are only two reports dealing with patients without interferences but one was retrospective (17) and the other evaluated the LT4 requirement in thyroidectomized patients (18). This study was designed to evaluate the potential effect of switching from tablet to softgel LT4, using serum TSH values as the main outcome. Therapeutic switch, at the same dose, involved patients certainly compliant with treatment without increased need for LT4.

## Materials and Methods

### Selection of Patients

**Figure 1:**
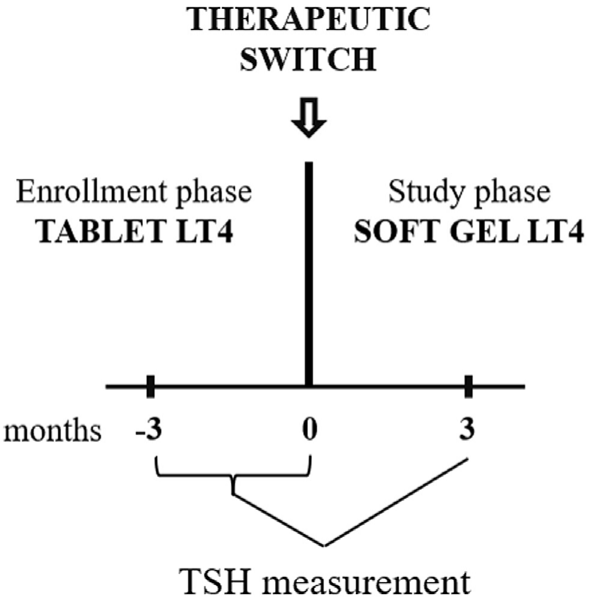
Diagram of the study design. In the enrollment phase, TSH was measured two times (−3 and 0 months). Patients were enrolled if the second value was similar to the first one. At 0 time point, all included patients were switched to softgel LT4 at unchanged dose. Following 3 months, TSH were re-evaluated and compared to the pre-switch value (the pre-switch value used has been the last one obtained).

According to the study aim, we enrolled hypothyroid patients on tablet LT4 therapy examined during the period from April 1 to August 31, 2017 (Enrollment Phase, see Figure 1). As inclusion criteria, patients must be hypothyroid in need for treatment with LT4 according to ATA Guidelines (1) due to autoimmune thyroiditis or total thyroidectomy for benign goiter. These patients were included regardless of their serum thyroid tests (i.e., serum TSH within the normal range or not) but showing serum TSH variations lesser than 1.0 mIU/L in two sequential control visits and they must be treated with LT4 in tablet formulation. We excluded patients with most of the described causes of malabsorption or increased requirement of LT4; then, were not enrolled patients with the following conditions either previous or active: presence and/or signs or symptoms suggestive for *H. pylori*-related gastritis (19), atrophic gastritis (20), celiac disease (21) or lactose intolerance (22), parasitic infestation (23), intestinal dysbiosis (24, 25), gastric, intestinal or bariatric surgery (26); pregnancy; concomitant treatment with PPIs, amiodarone, betablockers, lithium, raloxifene, interferons, cholestyramine, and antacids (7). We also looked for the presence of chronic unexplained anemia as sign of GI disorders (27). When only suspected for these conditions, patients underwent gastroenterological counseling. Finally, patients with neoplastic or chronic severe diseases were excluded because of the possibility of nonthyroidal illnesses. In all patients, according to the abovementioned criteria, we verified the compliance with LT4 therapy by specifically interviewing patients at every control visit; patients with suspected poor adherence to therapy (e.g., fluctuating TSH values in the two controls during enrollment, inconsistent answers to interview, etc.) were excluded. Local Ethical Committee does not require specific process for studies in which the authors use retrospective data of patients in anonymous and grouped manner; according to these rules, patients were informed orally on their possibility to be excluded from the study (i.e., right to object).

### Study Design

All patients were asked to switch from tablet to softgel LT4 formulation (Tirosint^®^ capsules molles, IBSA Institute Biochimique SA, CH-6903 Lugano, Switzerland) at unchanged dose and ingestion fashion (30 min before breakfast). Serum reflex TSH was re-evaluated 3 months after the switch (Study Phase, Figure 1). The pre-switch TSH used for comparison with the post-switch TSH is the one at 0 time point.

### Laboratory Measurement

Thyrotropin (TSH) was measured with chemiluminescence immunoassays (HYPERsensitive hTSH) on a UniCel DxI 800 automated platform (Beckman Coulter SA, Nyon, Switzerland). The HYPERsensitive hTSH assay is based on the third International Standard (WHO) for human TSH (IRP 81/565) with analytical sensitivity of 0.003 mIU/L, FS of 0.01 mIU/L, respectively. The TSH normal reference range is 0.40–4.00 mIU/L. Serum levels of free-T4 (normal range from 7.5 to 21.1 pmol/L), free-T3 (from 3.8 to 6.0 pmol/L), and TSH (from 0.4 to 4.0 mIU/mL) were automatically measured only in presence of skewed TSH value.

### Statistical Analysis

Value of TSH recorded on tablet and softgel LT4 were expressed as mean ± SD and compared by non-parametric statistical analysis for paired data (Wilcoxon test). Frequencies of patients with normal TSH with tablet and softgel LT4 were compared by chi square or Fisher’s exact test, when indicated. Statistical significance was set at *p* < 0.05. Statistical analysis was performed by GraphPad version 7 (GraphPad Prism, La Jolla CA, USA).

## Results

**Figure 2:**
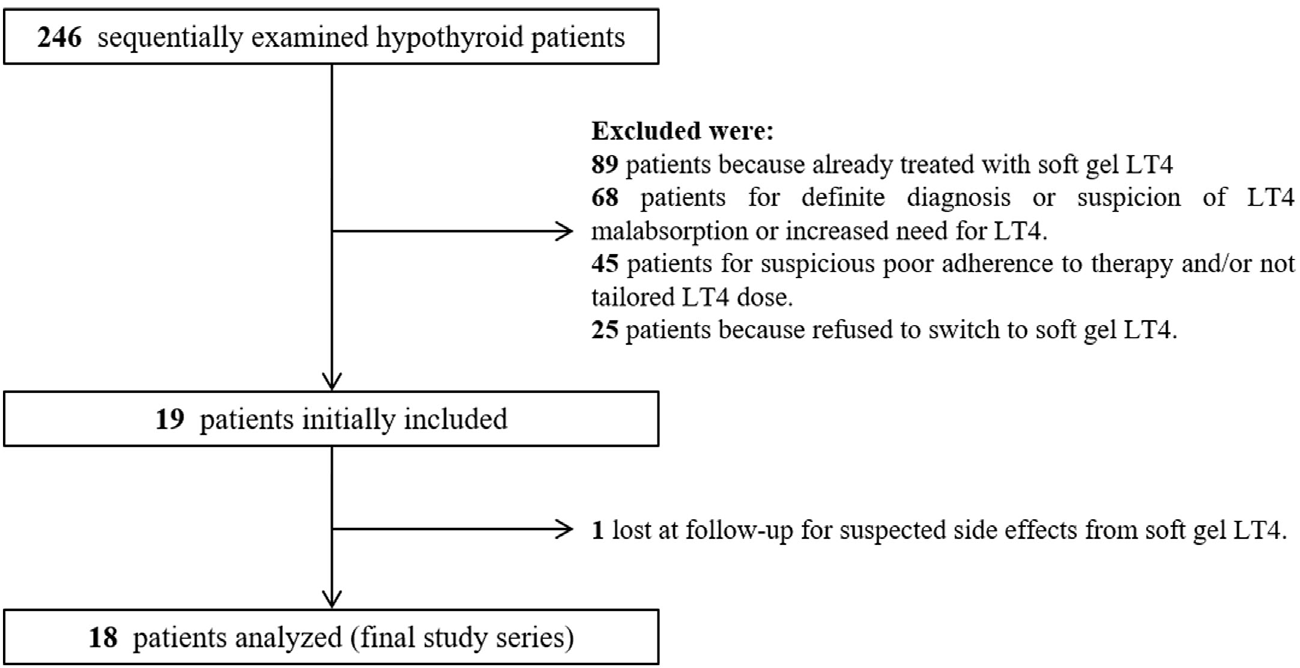
Diagram of flow of search strategy and results.

**Figure 3:**
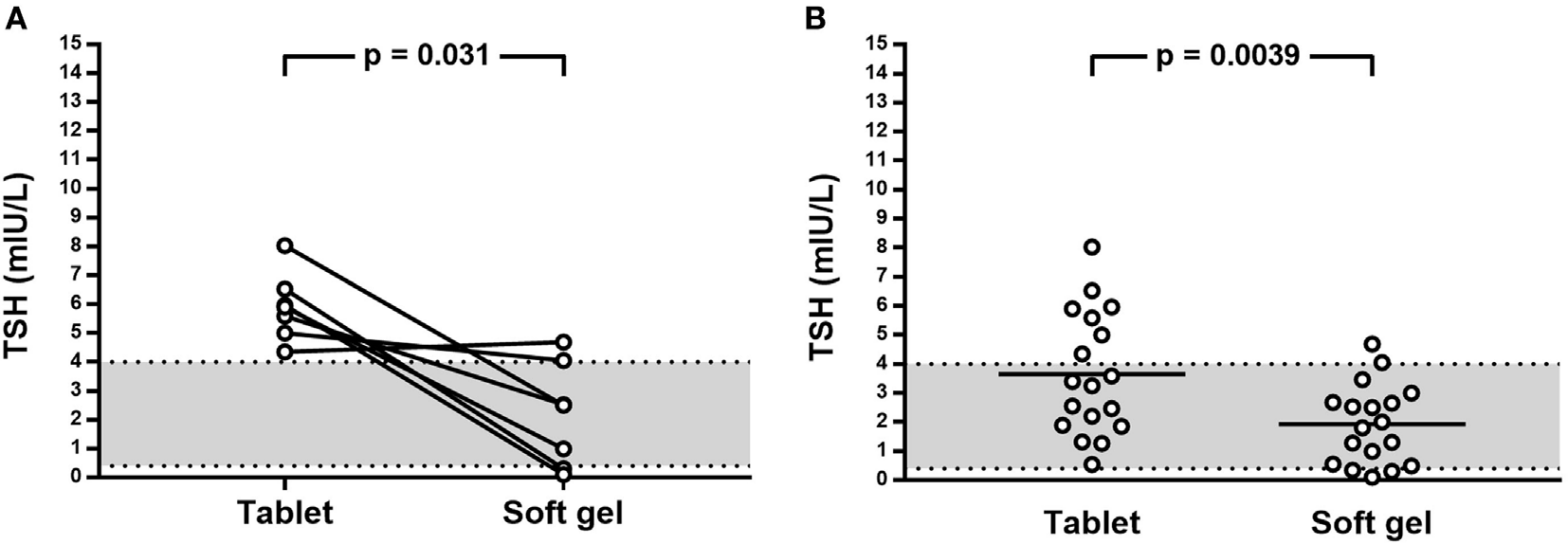
**(A)** Effect on TSH levels of switch from tablet to softgel LT4 preparation at the same dose in patient with TSH above 4.0 mIU/L; **(B)** Effect on TSH of switch from tablet to softgel LT4 preparation in the whole study series.

During the enrollment period, we examined 246 hypothyroid patients; among them, after initial selection, 19 (7.7%) patients met the inclusion criteria (Figure 2). All 19 patients were advised to switch from tablet to softgel LT4 at the same dose and with the same time schedule. One patient had headache four days later and come back to tablet LT4 with disappearance of headache. Then, 18 patients (16 females and 2 males, mean age 55 years; BMI 22.7 ± 2.9 kg/m^2^; 14 of them bearing Hashimoto’s thyroiditis and 4 thyroidectomized for benign goiter) represented the study group which completed the study. These patients were treated with a mean LT4 dose of 95.2 ± 23.5 μg/day (median 88 μg/day) and showed a mean TSH value of 3.65 ± 2.11 mIU/L (median 3.33 mIU/L, range from 0.54 to 8.03 mIU/L). The rate of patients with TSH within the normal reference range was 61.1% (11/18 cases). Serum FT4 and FT3 in those patients with increased TSH were anyway in the normal range. All cases were re-evaluated after 3 months of softgel LT4 therapy. During this period, no significant changes of BMI values (23.1 ± 3.1 kg/m^2^) and no side effects were recorded. Three months after the switch, we observed that TSH reached levels under 4.0 mIU/L in all patients but two who showed a serum TSH slightly higher than upper reference value (4.06 and 4.69 mIU/L) while in 3 patients, TSH was even lower than the reference range (0.10, 0.31, and 0.34 mIU/L). Normal free-T3 and free-T4 levels were again observed in all patients with TSH outside the normal range. Serum TSH was lower than the one before switch in 11 patients, similar in 5, including the two patients with serum TSH over 4.0 mIU/L, and higher in just one. Six out of seven patients with pre-switch TSH values over the normal range showed a lowered post-switch TSH value, in five of them lower than 4.0 mIU/L (Figure 3A).

Therefore, the number of patients with TSH lower than 4.0 mIU/L after the switch to softgel was 16/18 (88.9%); TSH values (mean 1.93 ± 1.36 mIU/L, median 1.90 mIU/L, range from 0.10 to 4.69 mIU/L) were significantly different from the one observed when patients were treated with tablet LT4 (*p* = 0.0039) being the mean difference 1.72 mIU/L (Figure 3B).

## Discussion

Several studies have shown that the absorption of LT4 in tablet formulation is impaired in various conditions such as gastric disorders, intestinal malabsorption, or drug interference, and this may be improved, in some cases, by switching to liquid LT4 preparation (9, 10). In addition, some studies showed that switching from tablet to liquid LT4 improved TSH levels of hypothyroid patients also in the absence of significant pathophysiological biases (28, 29). Furthermore, the study by Di Donna et al. demonstrated that LT4 requirement in tablet and softgel formulation is the same in thyroidectomized patients without malabsorption, despite a slightly reduced median TSH value in patients treated with softgel formulation (18). Here, we selected hypothyroid patients, mostly bearing Hashimoto’s thyroiditis, under levothyroxine therapy and without an increased need for thyroxine showing that the use of softgel LT4 significantly improved TSH values. Overall, lower TSH values were recorded in 4/5 of our patients and unchanged in the remaining. Owing to the role of gastric acid secretion on the process of T4 absorption (30), we may speculate that this finding may be due to the interindividual variation of gastric acidic output, even in gastric unaffected patients. A seminal pharmacokinetic study published in 2009 (31), has clearly established, *in vitro*, that softgel LT4 shows a better dissolution profile, at increasing pH values, as compared to tablet formulation. *In vivo*, using a single massive dose of 600 µg of labeled T4, softgel preparation, bioequivalent to tablet LT4 in fasting healthy volunteers, performed better than traditional formulation in patients with increased gastric pH (32). Moreover, this evidence has been clinically confirmed in patients with gastric diseases, in which target TSH had been reached with a reduced dose (−17%) in two-thirds of patients (12). The efficacy of this formulation has been also proven in patients with increased need for LT4 due to concomitant ingestion of coffee (11).

This study should have some limitations: first, we evaluated the two preparations performances in the very same way, spacing the drug and food or drink intake by 30 min, as suggested in the leaflet of tablet LT4. Evidence have been presented that this lag time may not interfere with the softgel preparation but may reduce significantly the absorption and then the effect of tablet T4 preparation (11, 33–37). This suggests the need for further studies, even with a more extended follow-up, in which all the parameters of thyroid pharmacological homeostasis are taken into account (i.e., comparing the efficacy of the two drugs by distancing at least an hour the intake of the drug and food). The consolidated habit, in both patients and physicians, to use a lag time of only 30 min before having breakfast prevented us from variating this schedule in the present study. Secondly, free-T4 and free-T3 values were not available for all patients because of the use of reflex TSH in clinical practice.

## Conclusion

These data show that hypothyroid patients with no proven malabsorption may have an improved TSH following 3 months from the switch from tablet to softgel LT4 preparation at unchanged dose. This evidence sheds light on the role that new formulations of levothyroxine can play to individualize the treatment in the era of precision medicine to limit the rate of patients with inadequate pharmacological thyroid homeostasis.

## Ethics Statement

This study was carried out in accordance with the Oncology Institute of Southern Switzerland.

## Author Contributions

PT and LG conceived and designed the study; PT and MC analyzed the data; CV, MC, and LG critically reviewed the results; CV and PT wrote the paper. All authors contributed to revise the manuscript.

## Conflict of Interest Statement

MC has been a consultant for Akrimax Pharmaceuticals, Cranford, NJ, USA, and received honoraria and travel expenses for participation in advisory boards, and from Institut Biochimique SA (IBSA), Lugano, CH for attending international meetings. The other authors have no conflict to declare. The reviewer SF and handling editor declared their shared affiliation.
